# Supplementary material for: Prevalence and determinants of anemia among Iranian population aged ≥35 years: A PERSIAN cohort–based cross-sectional study
Source: PLoS One. 2022 Feb 9;17(2):e0263795. doi: 10.1371/journal.pone.0263795 (PMC8827433; doi:10.1371/journal.pone.0263795)
Supplement: Attachment — Submitted filename: Response to Reviewers.docx [file pone.0263795.s001.docx]

**Authors’ Response to Reviewers’ Comments**

Journal title: PLOS ONE

Manuscript title: Prevalence and determinants of anemia among Iranian population aged ≥35 years: A PERSIAN cohort–based cross-sectional study

Manuscript Number: PONE-D-21-27694R1

Dear Dr Chenette and Prof Ashktorab,

Thank you very much for giving us the opportunity to submit a revised version of above manuscript to PLOS ONE journal. We would like to thank the editors and the reviewers for the time taken in reviewing and helping us to improve the paper. The reviewers’ comments have all been addressed. We attach a highlighted revised version of our paper with all alterations highlighted in yellow, a clean version, and a point-by-point response to the reviewers’ comments. All authors agree with its publication and confirm that it is not being considered for publication elsewhere.

Yours sincerely

On behalf of the authors

Journal Requirements:

When submitting your revision, we need you to address these additional requirements.

1. Please ensure that your manuscript meets PLOS ONE's style requirements, including those for file naming. The PLOS ONE style templates can be found at 
<https://journals.plos.org/plosone/s/file?id=wjVg/PLOSOne_formatting_sample_main_body.pdf> and 
<https://journals.plos.org/plosone/s/file?id=ba62/PLOSOne_formatting_sample_title_authors_affiliations.pdf>

Response: The authors appreciate the useful comments. The relevant corrections were made in the newer version of manuscript (font sizes, bibliography, etc.).

2. We note that you have indicated that data from this study are available upon request. PLOS only allows data to be available upon request if there are legal or ethical restrictions on sharing data publicly. For more information on unacceptable data access restrictions, please see <http://journals.plos.org/plosone/s/data-availability#loc-unacceptable-data-access-restrictions>.

In your revised cover letter, please address the following prompts:

a) If there are ethical or legal restrictions on sharing a de-identified data set, please explain them in detail (e.g., data contain potentially sensitive information, data are owned by a third-party organization, etc.) and who has imposed them (e.g., an ethics committee). Please also provide contact information for a data access committee, ethics committee, or other institutional body to which data requests may be sent.

b) If there are no restrictions, please upload the minimal anonymized data set necessary to replicate your study findings as either Supporting Information files or to a stable, public repository and provide us with the relevant URLs, DOIs, or accession numbers. For a list of acceptable repositories, please see <http://journals.plos.org/plosone/s/data-availability#loc-recommended-repositories>.

We will update your Data Availability statement on your behalf to reflect the information you provide.

Response: Thank you very much for your comment. The study protocol and individual participant data that underlie the results reported in this study, after de-identification (text, tables, and figures) can be shared with investigators whose proposed use of the data has been approved by the independent review committee of Tehran University of Medical Sciences and Digestive Diseases Research Institute. Data can be provided for projects related to the topic of the present study. The proposals should be directed to the PERSIAN cohort center (email: info@persiancohort.com), and/or Digestive Diseases Research Institute (email: info@ddri.ir), and/or Prof Reza Malekzadeh (email: dr.reza.malekzadeh@gmail.com), the senior author of the manuscript and the project leader.

3. PLOS requires an ORCID iD for the corresponding author in Editorial Manager on papers submitted after December 6th, 2016. Please ensure that you have an ORCID iD and that it is validated in Editorial Manager. To do this, go to ‘Update my Information’ (in the upper left-hand corner of the main menu), and click on the Fetch/Validate link next to the ORCID field. This will take you to the ORCID site and allow you to create a new iD or authenticate a pre-existing iD in Editorial Manager. Please see the following video for instructions on linking an ORCID iD to your Editorial Manager account: <https://www.youtube.com/watch?v=_xcclfuvtxQ>

Response: Thank you for your comment. The ORCID for the corresponding author has been verified.

4. We note that Figure 1 in your submission contain [map/satellite] images which may be copyrighted. All PLOS content is published under the Creative Commons Attribution License (CC BY 4.0), which means that the manuscript, images, and Supporting Information files will be freely available online, and any third party is permitted to access, download, copy, distribute, and use these materials in any way, even commercially, with proper attribution. For these reasons, we cannot publish previously copyrighted maps or satellite images created using proprietary data, such as Google software (Google Maps, Street View, and Earth). For more information, see our copyright guidelines: <http://journals.plos.org/plosone/s/licenses-and-copyright>.

We require you to either (1) present written permission from the copyright holder to publish these figures specifically under the CC BY 4.0 license, or (2) remove the figures from your submission:
 
a. You may seek permission from the original copyright holder of Figure 1 to publish the content specifically under the CC BY 4.0 license.

We recommend that you contact the original copyright holder with the Content Permission Form (<http://journals.plos.org/plosone/s/file?id=7c09/content-permission-form.pdf>) and the following text:
“I request permission for the open-access journal PLOS ONE to publish XXX under the Creative Commons Attribution License (CCAL) CC BY 4.0 (<http://creativecommons.org/licenses/by/4.0/>). Please be aware that this license allows unrestricted use and distribution, even commercially, by third parties. Please reply and provide explicit written permission to publish XXX under a CC BY license and complete the attached form.”
 
Please upload the completed Content Permission Form or other proof of granted permissions as an "Other" file with your submission.
 
In the figure caption of the copyrighted figure, please include the following text: “Reprinted from [ref] under a CC BY license, with permission from [name of publisher], original copyright [original copyright year].”
 
b. If you are unable to obtain permission from the original copyright holder to publish these figures under the CC BY 4.0 license or if the copyright holder’s requirements are incompatible with the CC BY 4.0 license, please either i) remove the figure or ii) supply a replacement figure that complies with the CC BY 4.0 license. Please check copyright information on all replacement figures and update the figure caption with source information. If applicable, please specify in the figure caption text when a figure is similar but not identical to the original image and is therefore for illustrative purposes only.
The following resources for replacing copyrighted map figures may be helpful:
 
USGS National Map Viewer (public domain): <http://viewer.nationalmap.gov/viewer/>
The Gateway to Astronaut Photography of Earth (public domain): <http://eol.jsc.nasa.gov/sseop/clickmap/>
Maps at the CIA (public domain): <https://www.cia.gov/library/publications/the-world-factbook/index.html> and <https://www.cia.gov/library/publications/cia-maps-publications/index.html>
NASA Earth Observatory (public domain): <http://earthobservatory.nasa.gov/>
Landsat: <http://landsat.visibleearth.nasa.gov/>
USGS EROS (Earth Resources Observatory and Science (EROS) Center) (public domain): [http://eros.usgs.gov/#](http://eros.usgs.gov/)
Natural Earth (public domain): <http://www.naturalearthdata.com/>

Response: We appreciate your comment. We designed the figures using Microsoft Office Excel and without plagiarism issue as mentioned in the methods, and we certify that no portion of this manuscript (text, figures) has been previously published.

5. Review Comments to the Author

Please use the space provided to explain your answers to the questions above. You may also include additional comments for the author, including concerns about dual publication, research ethics, or publication ethics. (Please upload your review as an attachment if it exceeds 20,000 characters)

Reviewer #1: This study was conducted in relatively large population to assess the prevalence of anemia and related probable risk facors in Iran.

My questions and recommendations are:

This manuscript is a result of a cross-sectional study. please change the title according to STROBE checklist. please specify the study age group (>=35 year) in title.

Response: Thank you very much for your comment. The title has been revised as per your recommendation.

Regarding sample size, power analysis in pure descriptive cross-sectional prevalence study is not mandatory. but in analytic C.S. study you should calculate the power of the study for comparisons.

Response: Thank you for the point. As per your comment, we have stated that the power of the study was approximately 1 based on the large sample size of the study and different anemia prevalence rates and odds ratios tested (Page 8).

Please explain about the method of sex and age standardization.

Response: We thank the reviewer’s comment. We have stated that the age- and sex-standardized prevalence rates were calculated using direct method, and Iran national census in 2016 were considered as a standard population (Page 8).

Please stated the version of STATA software.

Response: The version has been added.

Regarding the selection of confounding factors, according to which level of p- value you were selected them? what is the type of selection method? Step-wise method and p-value base criteria are not recommended.

Response: We appreciate your comment. We tried to select the potential determinants of anemia on the basis of the literature and availability in our database. Also, we only aimed to assess the association of the covariates with anemia, but not their predictive roles; therefore, we did not use step-wise method. We have mentioned new references in the revised manuscript upon which we chose the factors (Refs 9-11).

In table 1, because of your study were restricted to a special age group (35 years and older), please correct the age group strata. please calculate and show the amount of p-value for each variables. please clarify which level of category is different significantly? Also specify statistical test in each comparison. In the subtitle, which number in the table does refer to *p value?

Response: Thank you for your comment. The age group name has been corrected in all tables. We also added p-values for both of univariate and multivariable analyses in Table 3 as per your comment. A p-value less than 0.05 was considered significant.

How did you manage the missing data?

Response: The missing data were not included in the analyses. Considering that the rate of missing data was relatively very low, their effect would be ignorable.

In analyses of data from cross-sectional studies, the Poisson models with robust variance and calculating prevalence ratio(PR) are better alternatives than logistic regression and OR. Please use this analysis and compare the results with logistic regression analysis.

Response: Thank you for your comment. According to your recommendation, we have redone all of the relevant analyses and revised the main text and Table 3.

In table 4, specify the statistical tests in table subtitle. and stated which level of subgroup is different significantly.

Response: The statistical test (chi-squared test) and the level of significance have been added. We performed new analyses for each subgroup and added p-values in Table 4.

Discussion, please clarify all potential study bias in this study and your efforts to decrease them. for example, simultaneous measurement of exposure and outcome may lead to a very important bias and change the behavior of people after awareness of their disease. Were potential confounders identified? and were they managed appropriately in the study design and/or analysis? Please address the major potential cofounders according to literature. Please identify the effect measures are over- exaggerated or not? why? Discuss both direction and magnitude of any potential bias. Describe any efforts to address potential sources of bias.

Response: We thank you for your comments. About the simultaneous measurement of exposure and outcome, it should be noted that we did not enroll individuals to specifically assess their anemia only. In fact, we collected a list of data from the people for the PERSIAN cohort and one of the categories was hematologic data. Therefore, in the cohort, the individuals were informed not only about the protocol of hematologic data, but also about other variables at the same time. Thus, people were not aware of their disease during data collection. Regarding confounders, as replied earlier, we tried to select the high potential risk factors according to the literature and availability in our database; however, we agree with your concern that one of our limitations was lack of information on some influential factors, such as iron indices, vitamin levels, infections, or genetic disorders, to determine the etiology of anemia, which was mentioned in the limitations. Finally, we have mentioned that the present research was based on the respondents’ self-report and the collected data might have the likelihood of recall bias (Page 21).

The most important factor which is against the representative results of the your study is evaluation of anemia in a specific age group. How you can solve this limitation?

Response: According to your comment, we added this statement in the end of Discussion that we included adult subjects aged ≥35 years old and our interpretation on study representativeness should be based on this age group.

Reviewer #2: This is a good piece of research, but suffers from some mistakes in the statistical analysis that resulted in wrong interpretation of the result. In particular for logistic regression: 1) it is not clear whether all potential risk factors were included in the analysis

Response: Thank you very much for your comment. We tried to select the high potential risk factors according to the literature and availability in our database. In this regard, we chose 22 factors potentially associated with the risk of anemia; however, we agree with your concern that one of our limitations was lack of information on some influential factors, such as iron indices, vitamin levels, infections, or genetic disorders, to determine the etiology of anemia, which was mentioned in the Discussion as a limitation.

2) choice of reference group is wrong for some important factors like education and BMI. The reference group for education should be primary school and not "Illiterate''; similarly the reference category for BMI should be ''Normal'' and not underweight. This problem might happened for other risk factors that should be corrected. If you are careful in interpreting the result the current analysis could be OK. However, the conclusion stated that higher educational level increase the risk and higher BMI decrease the risk, these interpretations are inaccurate and misleading due to selection wrong reference category. In fact only underweight individuals are more at risk of anemia based on this data, which is logical. Regarding the education level if you select primary as your reference category then only illiterate will be significant, which you need to find interpretation for that by exploring other risk factors. You need to redo the analysis, that is why I suggested major revision otherwise it is rather few simple corrections in the statistical analysis followed by proper interpretation.

Response: We appreciate your helpful comment. We changed the reference category for “Residence”, “Educational level”, “BMI categories” and “Sleeping duration”, and re-analyzed the data (Table 3) and corrected the interpretations in the main text.
